# An Updated Meta-Analysis of Patent Foramen Ovale Closure Versus Medical Therapy for Preventing Recurrent Cryptogenic Stroke

**DOI:** 10.7759/cureus.97504

**Published:** 2025-11-22

**Authors:** Saif Almuzainy, Omar Hamodat, Rand Yahya, Salam Koniali, Rizwan Qaisar

**Affiliations:** 1 College of Medicine, University of Sharjah, Sharjah, ARE; 2 Basic Medical Sciences, College of Medicine, University of Sharjah, Sharjah, ARE; 3 Space Medicine Research Group, Research Institute of Medical and Health Sciences, University of Sharjah, Sharjah, ARE; 4 Cardiovascular Research Group, Research Institute of Medical and Health Sciences, University of Sharjah, Sharjah, ARE

**Keywords:** atrial fibrillation, cryptogenic stroke, medical therapy, patent foramen ovale, percutaneous pfo closure

## Abstract

The effectiveness of transcatheter patent foramen ovale (PFO) closure versus medical therapy for preventing recurrent strokes in cryptogenic stroke patients with PFO has been debated, with previous randomized controlled trials (RCTs) yielding mixed results. This meta-analysis was conducted to address these inconsistencies and include new evidence from a recently published RCT, offering updated insights into the efficacy and safety of these treatment strategies. We systematically searched PubMed, Scopus, and Ovid databases through December 2024. Eligible RCTs comparing PFO closure and medical therapy in cryptogenic stroke were included. The risk ratios (RR) with 95% confidence interval (CI) were computed, and P < 0.05 was considered as a level of significance. We performed subgroup analyses based on the presence of atrial septal aneurysm. Statistical analyses were performed using RevMan 5.3 (The Cochrane Collaboration, London, UK). Seven RCTs with 4,539 patients were included. PFO closure reduced recurrent stroke risk (RR: 0.39; 95% CI: 0.21-0.72; P = 0.003). No differences were found for transient ischemic attack (TIA), mortality, or bleeding. PFO closure increased the risk of new-onset atrial fibrillation (RR: 5.14; 95% CI: 2.93-9.01; P < 0.00001). Subgroup analysis showed a significant reduction in stroke with PFO closure in patients with an aneurysm (RR: 0.47; 95% CI: 0.26-0.88; P = 0.02), but not without (RR: 0.72; 95% CI: 0.47-1.11; P = 0.13). Transcatheter PFO closure demonstrates superior efficacy compared to medical therapy alone in reducing the risk of recurrent cryptogenic stroke.

## Introduction and background

Ischemic stroke is a leading cause of mortality and long-term disability worldwide. Despite advancements in diagnostic techniques, approximately 30-40% of ischemic strokes remain unexplained and are classified as cryptogenic strokes [1,2]. This subset of strokes presents a significant challenge in its management, as no clear etiology is identified.

A potential contributor to these strokes is a patent foramen ovale (PFO), a congenital defect characterized by the failure of the atrial septum to close completely after birth. It persists in roughly 25% of the general population, making it a common anatomical variant [3,4]. Numerous studies have shown that patients with cryptogenic stroke have a higher prevalence of PFO than patients with stroke of known etiology [5-7]. As a result, transcatheter closure of PFO has been proposed as a potential preventive strategy to reduce the risk of recurrent stroke in these patients.

The first reported case of transcatheter PFO closure occurred in 1992, and the procedure has since gained attention as a possible therapeutic option [8]. However, the effectiveness of PFO closure still remains controversial. While some meta-analyses have reported a reduction in recurrent stroke risk following PFO closure, others have yielded conflicting findings [9,10]. Moreover, concerns have been raised regarding potential complications, such as an increased incidence of atrial fibrillation following PFO closure. On the other hand, a recently published randomized controlled trial (RCT) by Liu et al. provided evidence supporting the superiority of PFO closure over medical therapy in preventing stroke recurrence, without a significant increase in the incidence of atrial fibrillation [11].

Given the emergence of new data and the ongoing debates surrounding the optimal management of patients with cryptogenic stroke and PFO, we conducted this updated meta-analysis to synthesize the latest evidence. Our goal is to compare the efficacy and safety of PFO closure versus medical therapy in preventing stroke recurrence, offering clinicians with updated insights to guide treatment decisions in this patient population.

## Review

Methodology

We followed the Preferred Reporting Items for Systematic Reviews and Meta-Analyses (PRISMA) statement guidelines during the preparation of this meta-analysis in reporting our methodology and findings.

Criteria for Considering Studies for This Review

For our meta-analysis, we applied the following inclusion criteria: (1) RCTs that investigated PFO closure with a percutaneous device as the intervention, compared to medical therapy (antiplatelet and/or anticoagulant therapy), (2) adult patients (age ≥18 years) with a history of ischemic neurological events, specifically stroke or transient ischemic attack (TIA), and a confirmed diagnosis of PFO, and (3) outcomes that include all-cause mortality, recurrent ischemic neurological events (stroke and TIA) occurring during the follow-up period, new-onset atrial fibrillation, and major bleeding events. We excluded studies that did not meet these inclusion criteria, as well as animal studies, non-English studies, case reports, case series, editorials, reviews, and non-randomized or unpublished studies.

Search Strategy

To identify relevant studies, comprehensive searches were conducted in PubMed, Scopus, and Ovid databases up to the date of manuscript preparation. When the same patient population was included in several publications, only the most recent or complete study would be included.

The search strategy utilized a combination of specific keywords and Medical Subject Headings (MeSH) terms aligned with our study objectives. The search terms included the following: "Patent Foramen Ovale", "PFO Closure", "Percutaneous PFO Closure", "Percutaneous Closure", "Medical Therapy", "Anticoagulant Therapy", "Antiplatelet Therapy", "Cryptogenic Stroke", "Stroke", "Transient Ischemic Attack", and "Recurrent Neurological Events."

Selection of Studies

Two authors (S.A. and O.H.) independently applied the inclusion and exclusion criteria to all the records. Screening was performed in a two-step process: The first step was to screen titles and abstracts of the retrieved studies for relevance, and in the second step, full texts of potentially eligible studies were reviewed for inclusion. Disagreements were resolved through discussion. Additionally, references of relevant publications were manually reviewed to ensure the comprehensive inclusion of all potential studies of interest, identifying any additional relevant research that may not have been retrieved through database searches.

Data Extraction

Four authors extracted the data independently using an online data extraction form. The extracted data fell under the following categories: (1) study characteristics (study design, year of publication, randomization, types of closure devices used, types of medical therapy used in each arm, follow-up period, and primary outcomes), (2) baseline population characteristics (main demographic characteristics and disease history), (3) quality assessment using the Cochrane Risk of Bias (ROB 1) tool, and (4) event counts for each outcome (all-cause mortality, recurrent ischemic neurological events (stroke and TIA), new-onset atrial fibrillation, and major bleeding events).

Quality Assessment of the Included Studies

Two authors independently assessed the quality of the included RCTs using the Cochrane ROB 1 tool. The assessment focused on seven key domains including random sequence generation, allocation concealment, blinding of participants and personnel, blinding of outcome assessment, incomplete outcome data, selective reporting, and other sources of bias. We graded these items as having high, low, or unclear risk.

Data Analysis and Synthesis

Continuous variables are presented as mean ± standard deviation (SD), while categorical variables are shown as n (%). Meta-analyses were performed using the risk ratio (RR) for categorical variables. Variables reported by two or more studies were pooled. Statistical pooling was conducted using the Mantel-Haenszel method for categorical variables; a P-value of <0.05 was assigned as the measure of statistical significance.

Heterogeneity was assessed through the visual inspection of the forest plots and quantified using I-squared and chi-squared tests. A significant heterogeneity was detected if the chi-squared P-value is <0.10. A random-effects model was applied when outcomes showed heterogeneity to account for potential variation in methodology and participant characteristics between studies; otherwise, a fixed-effects model was used. Subgroup analyses were performed based on the presence of atrial septal aneurysm. RRs with 95% confidence intervals (CIs) were computed using RevMan 5.3 (The Cochrane Collaboration, London, UK).

Publication Bias

It was not possible to assess publication bias due to the relatively small number of included studies (<10) [12].

Results

Study Selection

**Figure 1:**
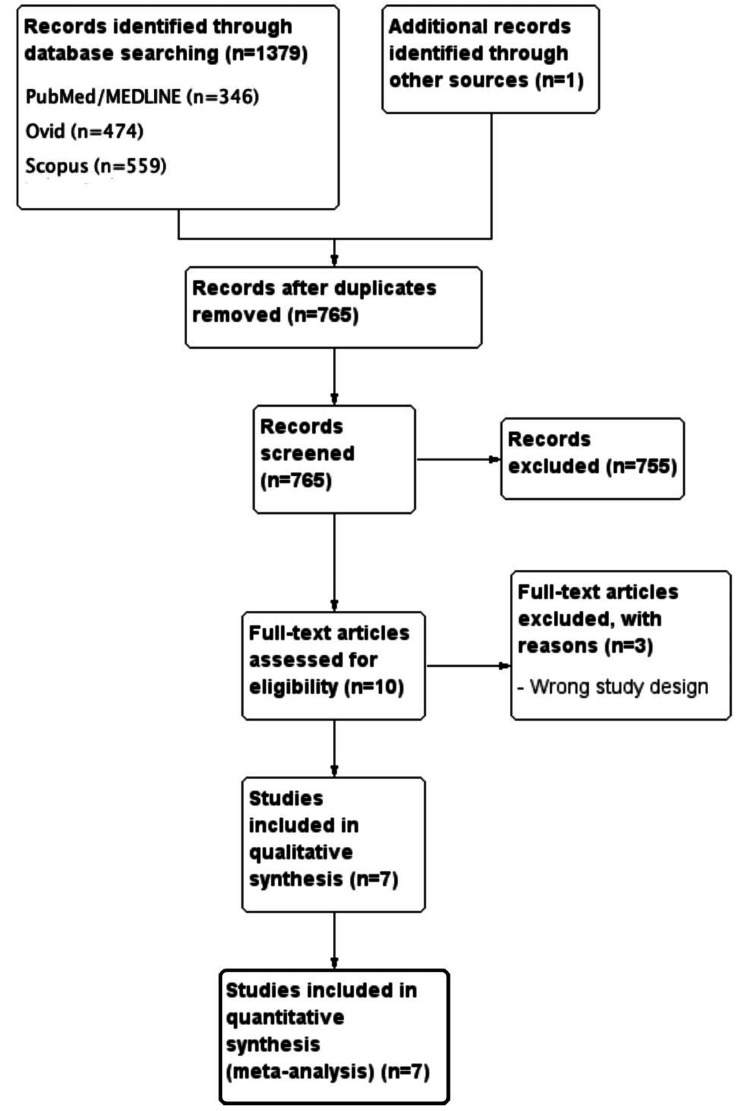
PRISMA study flow diagram PRISMA: Preferred Reporting Items for Systematic Reviews and Meta-Analyses

A comprehensive search yielded 1,379 records, including 346 from PubMed/MEDLINE, 474 from Ovid, and 559 from Scopus, along with one additional record from other sources. After duplicate removal, 765 unique records were available for screening. Of these, 755 were excluded based on title and abstract review. The full texts of 10 articles were evaluated for eligibility, with three excluded due to unsuitable study designs. Ultimately, seven studies met the inclusion criteria and were incorporated into the meta-analysis. The study selection process is detailed in the PRISMA flow diagram (Figure 1).

Study Characteristics

Seven studies were included in this meta-analysis, with their key characteristics summarized in Table 1.

**Table 1:** Characteristics of the included studies ^a^PFO closure:AP alone:AC. ^b^PFO closure:AP. CLOSE: Patent Foramen Ovale Closure or Anticoagulants Versus Antiplatelet Therapy to Prevent Stroke Recurrence; RESPECT: Randomized Evaluation of Recurrent Stroke Comparing PFO Closure to Established Current Standard of Care Treatment; PC Trial: Clinical Trial Comparing Percutaneous Closure of Patent Foramen Ovale Using the Amplatzer PFO Occluder With Medical Treatment in Patients With Cryptogenic Embolism; CLOSURE I: Evaluation of the STARFlex Septal Closure System in Patients With a Stroke and/or Transient Ischemic Attack due to Presumed Paradoxical Embolism Through a Patent Foramen Ovale; DEFENSE-PFO: Device Closure Versus Medical Therapy for Cryptogenic Stroke Patients With High-Risk Patent Foramen Ovale; AC: anticoagulation; AP: antiplatelet; DAPT: dual antiplatelet therapy; INR: international normalized ratio; PFO: patent foramen ovale; TIA: transient ischemic attack; TIMI: thrombolysis in myocardial infarction; MT: medical therapy

Study ID	Enrollment	Randomization (PFO closure:MT)	Intervention	PFO device type	MT	Follow-up, years	Primary outcome
CLOSE[13]	2008-2014; multicenter, randomized	1:1:1^a^	PFO closure with DAPT for 3 months and then single AP for the rest of the trial	Multiple (Amplatzer 51.5%)	AC: vitamin K antagonist (INR 2-3) or DOAC. AP: aspirin or clopidogrel or aspirin + dipyridamole for the rest of the trial	5.3	Fatal or nonfatal stroke
CLOSURE I[14]	2003-2008; multicenter, randomized	1:1	PFO closure + antiplatelet regimen including clopidogrel for 6 months and aspirin for 2 years	STARFlex	Warfarin, aspirin, or both	2	Composite of stroke or TIA during 2 years of follow-up, death from any cause during the first 30 days, or death from neurologic causes between 31 days and 2 years
DEFENSE-PFO [15]	2011-2017; multicenter, randomized	1:1	Amplatzer PFO occluder + aspirin AND clopidogrel for at least 6 months	Amplatzer	Single or DAPT or AC (warfarin)	2.8	Composite of stroke, vascular death, or TIMI-defined major bleeding
PC Trial[16]	2000-2009; multicenter, randomized	1:1	Amplatzer PFO occluder + aspirin for at least 5-6 months AND ticlopidine OR clopidogrel or 1-6 months	Amplatzer	AP therapy or AC	4.1	Composite of death, nonfatal stroke, TIA, or peripheral embolism
REDUCE[17]	2008-2015; multicenter, randomized	2:1^b^	PFO closure with clopidogrel for 3 days, followed by an AP for the rest of the trial	HELEX or cardioform	Aspirin (75-325 mg), aspirin + dipyridamole, or clopidogrel	3.2	Clinical stroke and new brain infarction
RESPECT[18]	2003-2011; multicenter, randomized	1:1	Amplatzer PFO occluder + aspirin and clopidogrel for 1 month, followed by aspirin for at least 5 months	Amplatzer	Aspirin, clopidogrel, warfarin, or aspirin + dipyridamole	5.9	Composite of recurrent nonfatal stroke, fatal stroke, or early death
Liu et al. 2021 [11]	2013-2018; multicenter, randomized	1:1	Implantation of PFO occluders + aspirin for 6 months and clopidogrel for 3 months	LifeTech PFO Occluder	Aspirin as an AP drug or warfarin as an AC drug	3.6	Stroke recurrence and TIA

The analysis incorporated data from 4,539 patients across seven RCTs, with a mean age of 43.6 years and a male predominance of 53.38%. Baseline characteristics were well-balanced between the PFO closure and medical therapy groups. Comorbidities showed comparable prevalence, including hypertension (24.37% vs. 25.28%), hyperlipidemia (27.37% vs. 29.45%), diabetes mellitus (4.81% vs. 6.29%), smoking (20.87% vs. 20.96%), migraine (30.10% vs. 29.93%), and prior stroke (45.83% vs. 46.24%). Similar distributions were observed for moderate or greater shunts (66.68% vs. 62.23%), atrial septal aneurysm (24.22% vs. 25.72%), and deep vein thrombosis/pulmonary embolism (DVT/PE) (2.60% vs. 1.84%). Further demographic and baseline details are outlined in Table 2.

**Table 2:** Baseline clinical characteristics in the PFO closure versus medical therapy groups Data are n, mean ± SD, median (range), or n (%). CLOSE: Patent Foramen Ovale Closure or Anticoagulants Versus Antiplatelet Therapy to Prevent Stroke Recurrence; RESPECT: Randomized Evaluation of Recurrent Stroke Comparing PFO Closure to Established Current Standard of Care Treatment; PC Trial: Clinical Trial Comparing Percutaneous Closure of Patent Foramen Ovale Using the Amplatzer PFO Occluder With Medical Treatment in Patients With Cryptogenic Embolism; CLOSURE I: Evaluation of the STARFlex Septal Closure System in Patients With a Stroke and/or Transient Ischemic Attack due to Presumed Paradoxical Embolism Through a Patent Foramen Ovale; DEFENSE-PFO: Device Closure Versus Medical Therapy for Cryptogenic Stroke Patients With High-Risk Patent Foramen Ovale; PFO: patent foramen ovale; DVT: deep vein thrombosis; PE: pulmonary embolism

Study ID	Group	Sample size	Male, N (%)	Age (years)	Hypertension	Hyperlipidemia	Diabetes	Smoking	Migraine	Previous stroke	Moderate or higher shunt	Atrial septal aneurysm	DVT/PE
RESPECT [18]	PFO closure	499	268 (53.7)	45.7 ± 9.7	158 (31.7)	194 (38.9)	33 (6.6)	75 (15)	195 (39.1)	53 (10.6)	385 (78)	180 (36.1)	20 (4.0)
	Medical therapy	481	268 (55.7)	46.2 ± 10.0	150 (31.2)	193 (40.1)	40 (8.3)	55 (11.4)	185 (38.5)	51 (10.6)	352 (72)	169 (35.1)	15 (3.1)
REDUCE[17]	PFO closure	664	261 (59.2)	45.4 ± 9.3	112 (25.4)	-	18 (4.1)	63 (14.3)	-	62 (14.1)	348 (81.8)	86 (20.4)	-
	Medical therapy	233	138 (61.9)	44.8 ± 9.6	58 (26.0)	-	10 (4.5)	25 (11.2)	-	23 (10.3)	173 (80)	-	-
CLOSE[13]	PFO closure	238	137 (57.6)	42.9 ± 10.1	27 (11.3)	30 (12.6)	3 (1.3)	68 (28.6)	67 (28.2)	10 (4.2)	179 (75.2)	-	5 (2.1)
	Medical therapy	422	142 (60.4)	43.8 ± 10.5	24 (10.2)	36 (15.3)	9 (3.8)	69 (29.4)	78 (33.2)	7 (3.0)	173 (73.6)	-	4 (1.7)
CLOSURE I [14]	PFO closure	447	233 (52.1)	46.3 ± 9.6	151 (33.8)	212 (47.4)		96 (21.5)	-	324 (72.6)	250 (55.9)	168 (37.6)	0
	Medical therapy	462	238 (51.5)	45.7 ± 9.1	131 (28.4)	189 (40.9)		104 (22.6)	-	329 (71.4)	231 (50.0)	165 (35.7)	4 (0.9)
PC Trial[16]	PFO closure	204	92 (45.1)	44.3 ± 10.2	49 (24.0)	50 (24.5)	5 (2.5)	52 (25.5)	47 (23.0)	165 (80.9)	130 (70.2)	47 (23.0)	6 (2.9)
	Medical therapy	210	114 (54.3)	44.6 ± 10.1	58 (27.6)	62 (29.5)	6 (2.9)	47 (22.4)	38 (18.1)	163 (77.6)	112 (60.9)	51 (24.3)	5 (2.4)
DEFENSE-PFO[15]	PFO closure	60	33 (55.0)	49 ± 15	12 (20.0)	18 (30.0)	6 (10.0)	10 (16.7)	-	28 (46.7)	-	5 (8.3)	-
	Medical therapy	60	34 (56.7)	54 ± 12	17 (28.3)	25 (41.7)	8 (13.3)	16 (26.7)	-	36 (60.0)	-	8 (13.3)	-
Liu et al. 2021[11]	PFO closure	277	116 (41.9)	30.2 ± 9.7	-	30 (10.8)	12 (4.33)	68 (24.5)	-	254 (91.7)	108 (39)	55 (19.9)	4 (1.4)
	Medical therapy	282	119 (42.2)	31.3 ± 10.1	-	26 (9.2)	14 (4.96)	65 (23)	-	256 (90.8)	104 (36.9)	57 (20.2)	3 (1.1)

Risk of Bias in Studies

**Figure 2:**
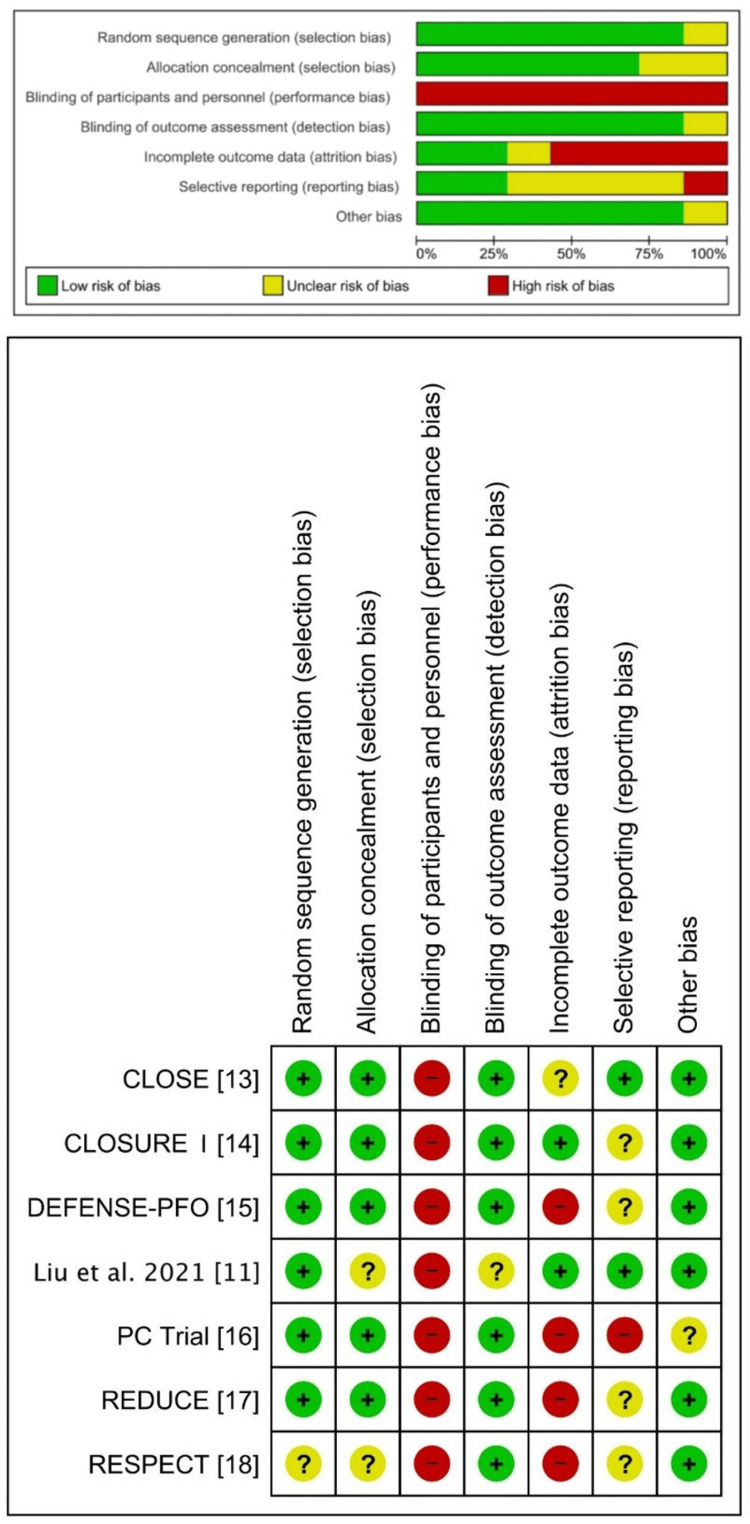
The Cochrane ROB 1 quality assessment of the included studies CLOSE: Patent Foramen Ovale Closure or Anticoagulants Versus Antiplatelet Therapy to Prevent Stroke Recurrence; RESPECT: Randomized Evaluation of Recurrent Stroke Comparing PFO Closure to Established Current Standard of Care Treatment; PC Trial: Clinical Trial Comparing Percutaneous Closure of Patent Foramen Ovale Using the Amplatzer PFO Occluder With Medical Treatment in Patients With Cryptogenic Embolism; CLOSURE I: Evaluation of the STARFlex Septal Closure System in Patients With a Stroke and/or Transient Ischemic Attack due to Presumed Paradoxical Embolism Through a Patent Foramen Ovale; DEFENSE-PFO: Device Closure Versus Medical Therapy for Cryptogenic Stroke Patients With High-Risk Patent Foramen Ovale; ROB: Risk of Bias

The Cochrane ROB 1 tool revealed a low-to-moderate risk of bias across the seven included studies. A comprehensive summary of the risk of bias for each study is illustrated in Figure 2.

Pooled Results

**Figure 3:**
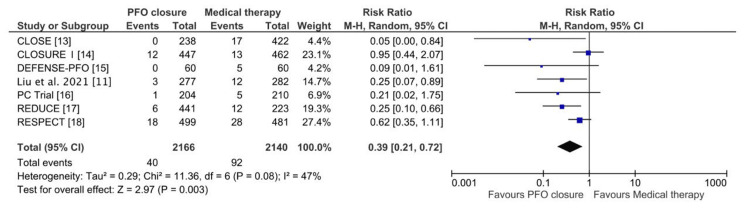
Forest plot of risk ratios of stroke attacks between the PFO closure and medical therapy groups CLOSE: Patent Foramen Ovale Closure or Anticoagulants Versus Antiplatelet Therapy to Prevent Stroke Recurrence; RESPECT: Randomized Evaluation of Recurrent Stroke Comparing PFO Closure to Established Current Standard of Care Treatment; PC Trial: Clinical Trial Comparing Percutaneous Closure of Patent Foramen Ovale Using the Amplatzer PFO Occluder With Medical Treatment in Patients With Cryptogenic Embolism; CLOSURE I: Evaluation of the STARFlex Septal Closure System in Patients With a Stroke and/or Transient Ischemic Attack due to Presumed Paradoxical Embolism Through a Patent Foramen Ovale; DEFENSE-PFO: Device Closure Versus Medical Therapy for Cryptogenic Stroke Patients With High-Risk Patent Foramen Ovale; CI: confidence interval; M-H: Mantel-Haenszel; PFO: patent foramen ovale

**Figure 4:**
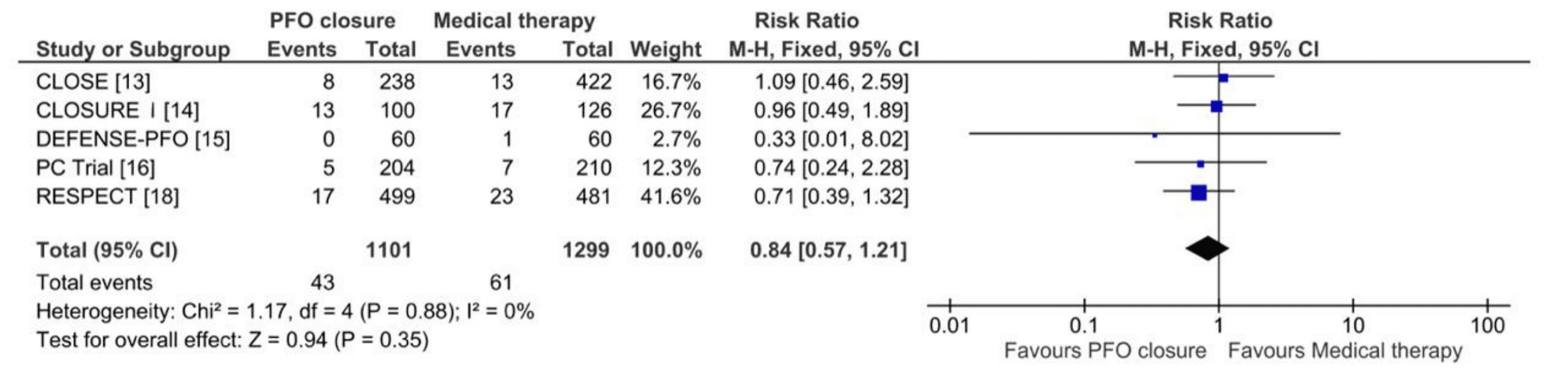
Forest plot of pooled risk ratios comparing transient ischemic attacks between the PFO closure and medical therapy groups CLOSE: Patent Foramen Ovale Closure or Anticoagulants Versus Antiplatelet Therapy to Prevent Stroke Recurrence; RESPECT: Randomized Evaluation of Recurrent Stroke Comparing PFO Closure to Established Current Standard of Care Treatment; PC Trial: Clinical Trial Comparing Percutaneous Closure of Patent Foramen Ovale Using the Amplatzer PFO Occluder With Medical Treatment in Patients With Cryptogenic Embolism; CLOSURE I: Evaluation of the STARFlex Septal Closure System in Patients With a Stroke and/or Transient Ischemic Attack due to Presumed Paradoxical Embolism Through a Patent Foramen Ovale; DEFENSE-PFO: Device Closure Versus Medical Therapy for Cryptogenic Stroke Patients With High-Risk Patent Foramen Ovale; CI: confidence interval; M-H: Mantel-Haenszel; PFO: patent foramen ovale

This meta-analysis evaluates the efficacy of PFO closure versus medical therapy alone in reducing the risk of recurrent stroke. A total of 2166 patients underwent PFO closure, while 2140 received medical therapy alone. The pooled RR for recurrent stroke significantly favoured PFO closure, with an RR of 0.39 (95% CI: 0.21-0.72; P = 0.003), indicating a 61% relative risk reduction, as shown in Figure 3. Moderate heterogeneity was observed across studies (Chi² = 11.36; I² = 47%; P = 0.08), and a random-effects model was applied. Moreover, for TIA, 1101 patients underwent PFO closure, while 1299 were treated with medical therapy alone. The pooled RR was 0.84 (95% CI: 0.57-1.21; P = 0.35), as shown in Figure 4, indicating no significant difference between the two treatment strategies. Heterogeneity was minimal (Chi² = 1.17; I² = 0%; P = 0.88), and a fixed-effects model was applied.

**Figure 5:**
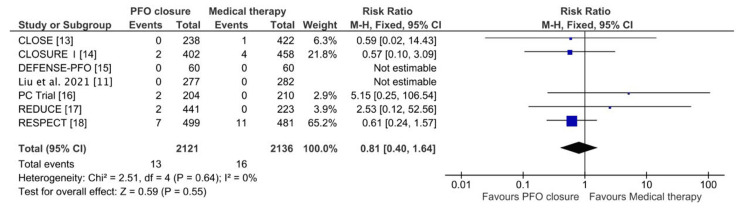
Forest plot of pooled risk ratios showing the comparison of all-cause mortality between the PFO closure and medical therapy groups CLOSE: Patent Foramen Ovale Closure or Anticoagulants Versus Antiplatelet Therapy to Prevent Stroke Recurrence; RESPECT: Randomized Evaluation of Recurrent Stroke Comparing PFO Closure to Established Current Standard of Care Treatment; PC Trial: Clinical Trial Comparing Percutaneous Closure of Patent Foramen Ovale Using the Amplatzer PFO Occluder With Medical Treatment in Patients With Cryptogenic Embolism; CLOSURE I: Evaluation of the STARFlex Septal Closure System in Patients With a Stroke and/or Transient Ischemic Attack due to Presumed Paradoxical Embolism Through a Patent Foramen Ovale; DEFENSE-PFO: Device Closure Versus Medical Therapy for Cryptogenic Stroke Patients With High-Risk Patent Foramen Ovale; CI: confidence interval; M-H: Mantel-Haenszel; PFO: patent foramen ovale

Similarly, for all-cause mortality, 2121 patients underwent PFO closure, while 2136 received medical therapy alone. The pooled RR was 0.81 (95% CI: 0.40-1.64; P = 0.55), as shown in Figure 5, indicating no significant difference between the two groups. Heterogeneity was negligible (Chi² = 2.51; I² = 0%; P = 0.64), and a fixed-effects model was applied, suggesting that PFO closure does not significantly impact all-cause mortality compared to medical therapy alone.

**Figure 6:**
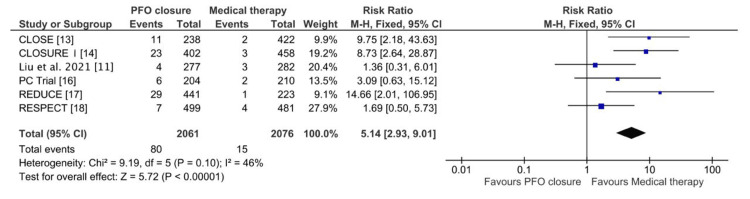
Forest plot of pooled effect estimates comparing new-onset atrial fibrillation between the PFO closure and medical therapy groups CLOSE: Patent Foramen Ovale Closure or Anticoagulants Versus Antiplatelet Therapy to Prevent Stroke Recurrence; RESPECT: Randomized Evaluation of Recurrent Stroke Comparing PFO Closure to Established Current Standard of Care Treatment; PC Trial: Clinical Trial Comparing Percutaneous Closure of Patent Foramen Ovale Using the Amplatzer PFO Occluder With Medical Treatment in Patients With Cryptogenic Embolism; CLOSURE I: Evaluation of the STARFlex Septal Closure System in Patients With a Stroke and/or Transient Ischemic Attack due to Presumed Paradoxical Embolism Through a Patent Foramen Ovale; CI: confidence interval; M-H: Mantel-Haenszel; PFO: patent foramen ovale

In contrast, for new-onset atrial fibrillation, 2061 patients underwent PFO closure, while 2076 received medical therapy alone. The pooled RR was 5.14 (95% CI: 2.93-9.01; P < 0.00001), as shown in Figure 6, indicating a significantly higher risk of atrial fibrillation associated with PFO closure. Moderate heterogeneity was observed (Chi² = 9.19; I² = 46%; P = 0.10), and a fixed-effects model was applied. These findings highlight the need to weigh the benefits of PFO closure in preventing recurrent stroke against its associated risk of atrial fibrillation.

**Figure 7:**
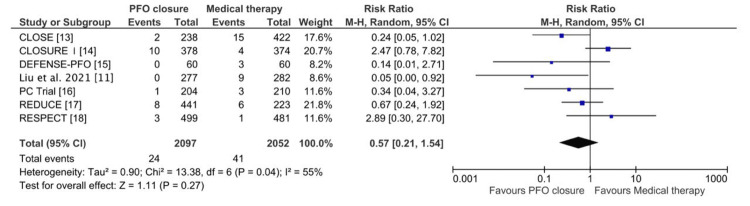
Forest plot of pooled risk ratios comparing major bleeding events between the PFO closure and medical therapy groups CLOSE: Patent Foramen Ovale Closure or Anticoagulants Versus Antiplatelet Therapy to Prevent Stroke Recurrence; RESPECT: Randomized Evaluation of Recurrent Stroke Comparing PFO Closure to Established Current Standard of Care Treatment; PC Trial: Clinical Trial Comparing Percutaneous Closure of Patent Foramen Ovale Using the Amplatzer PFO Occluder With Medical Treatment in Patients With Cryptogenic Embolism; CLOSURE I: Evaluation of the STARFlex Septal Closure System in Patients With a Stroke and/or Transient Ischemic Attack due to Presumed Paradoxical Embolism Through a Patent Foramen Ovale; DEFENSE-PFO: Device Closure Versus Medical Therapy for Cryptogenic Stroke Patients With High-Risk Patent Foramen Ovale; CI: confidence interval; M-H: Mantel-Haenszel; PFO: patent foramen ovale

Finally, for bleeding events, 2097 patients underwent PFO closure, while 2052 received medical therapy alone. The pooled RR was 0.57 (95% CI: 0.21-1.54; P = 0.27), as shown in Figure 7, indicating no significant difference between the two groups. Moderate heterogeneity was noted (Chi² = 13.38; I² = 55%; P = 0.04), and a random-effects model was applied, suggesting that PFO closure does not significantly alter the risk of bleeding events compared to medical therapy alone.

Subgroup Analysis

**Figure 8:**
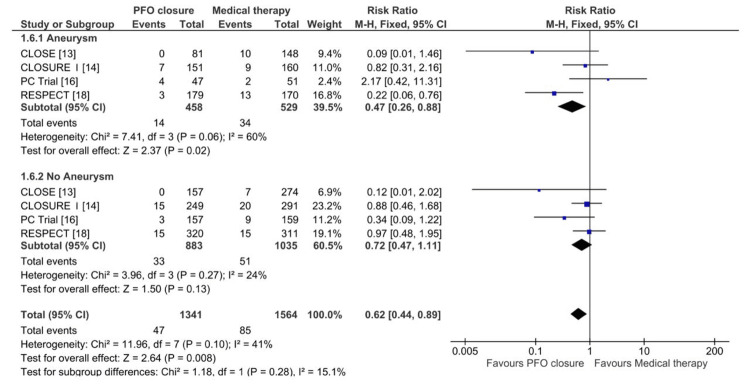
Forest plot of pooled risk ratios comparing stroke attacks between the PFO closure and medical therapy groups among patients with and without atrial septal aneurysm CLOSE: Patent Foramen Ovale Closure or Anticoagulants Versus Antiplatelet Therapy to Prevent Stroke Recurrence; RESPECT: Randomized Evaluation of Recurrent Stroke Comparing PFO Closure to Established Current Standard of Care Treatment; PC Trial: Clinical Trial Comparing Percutaneous Closure of Patent Foramen Ovale Using the Amplatzer PFO Occluder With Medical Treatment in Patients With Cryptogenic Embolism; CLOSURE I: Evaluation of the STARFlex Septal Closure System in Patients With a Stroke and/or Transient Ischemic Attack due to Presumed Paradoxical Embolism Through a Patent Foramen Ovale; CI: confidence interval; M-H: Mantel-Haenszel; PFO: patent foramen ovale

This meta-analysis also evaluates the impact of PFO closure versus medical therapy alone on the risk of recurrent stroke in patients with and without an associated aneurysm. In the subgroup with an aneurysm, PFO closure significantly reduced the risk of recurrent stroke, with a pooled RR of 0.47 (95% CI: 0.26-0.88; P = 0.02), as depicted in Figure 8. Moderate heterogeneity was observed (Chi² = 7.41; I² = 60%; P = 0.06), and a fixed-effects model was applied. Conversely, in the subgroup without an aneurysm, no significant benefit was observed with PFO closure, with a pooled RR of 0.72 (95% CI: 0.47-1.11; P = 0.13), and minimal heterogeneity was present (Chi² = 3.96; I² = 24%; P = 0.27). The overall pooled analysis across both subgroups revealed a significant reduction in recurrent stroke risk with PFO closure (RR: 0.62; 95% CI: 0.44-0.89; P = 0.008), with moderate heterogeneity (Chi² = 11.96; I² = 41%; P = 0.10). These findings indicate that the presence of an aneurysm may modify the efficacy of PFO closure in preventing recurrent stroke.

Discussion

This meta-analysis comprehensively evaluated the efficacy and safety of PFO closure versus medical therapy alone in patients with cryptogenic stroke. The findings offer valuable insights into clinical decision-making for this population, highlighting the trade-offs between stroke prevention and potential adverse effects.

Key Findings and Implications

Our findings consolidate the results of previous RCTs comparing device closure and medical therapy for cryptogenic stroke [11,13-18]. The pooled results demonstrate that PFO closure significantly reduces the risk of recurrent stroke compared to medical therapy alone (RR: 0.39; 95% CI: 0.21-0.72; P = 0.003), indicating a 61% relative risk reduction. This robust evidence underscores the efficacy of PFO closure in secondary stroke prevention. The presence of moderate heterogeneity (I² = 47%) suggests that variations in study design, patient selection, or device types might partially influence the observed effect size. These findings are consistent with prior studies, including meta-analyses [19,20], which similarly highlighted the efficacy of PFO closure in preventing recurrent stroke among patients with high-risk PFO characteristics.

However, the analysis revealed no significant differences between the two treatment strategies in reducing TIAs, all-cause mortality, or bleeding events. These findings suggest that while PFO closure excels in preventing recurrent strokes, it may not confer additional benefits in other outcomes. This is consistent with the results of another meta-analysis by Pan et al. [21], which found insufficient evidence to support PFO closure over medical therapy for the secondary prevention of cryptogenic stroke.

Importantly, the risk of new-onset atrial fibrillation was significantly higher in the PFO closure group (RR: 5.14; 95% CI: 2.93-9.01; P < 0.00001). This result emphasizes the need for careful patient selection and monitoring, as the increased incidence of atrial fibrillation could potentially offset the benefits of stroke reduction. Moderate heterogeneity (I² = 46%) warrants further exploration of patient- or procedure-specific factors contributing to this risk. Similar observations were documented by Garg et al. and Ahmad et al. [19,20], who reported a heightened incidence of atrial fibrillation following PFO closure. These findings underscore the procedural risk of atrial fibrillation and highlight its significant implications for clinical decision-making and patient management.

Subgroup Analysis

The subgroup analysis revealed that the efficacy of PFO closure in reducing recurrent stroke was more pronounced in patients with an associated atrial septal aneurysm (RR: 0.47; 95% CI: 0.26-0.88; P = 0.02). Conversely, in patients without an aneurysm, no significant benefit was observed (RR: 0.72; 95% CI: 0.47-1.11; P = 0.13). These findings highlight the potential role of aneurysms as effect modifiers, suggesting that patients with this anatomical variation may derive greater benefit from PFO closure. These findings align with the conclusions of Garg et al. [19], who emphasized the pivotal role of high-risk PFO features, including atrial septal aneurysm and large shunt size, in guiding therapeutic strategies. Likewise, Saver et al. [18] reinforced the importance of anatomical characteristics in shaping clinical decision-making.

Clinical and Research Implications

The results of this meta-analysis highlight the need for individualized decision-making when considering PFO closure in patients with cryptogenic stroke. While the intervention offers significant protection against recurrent stroke, the associated risks, particularly atrial fibrillation, necessitate a balanced approach. Multidisciplinary teams should weigh the benefits and risks, considering patient-specific factors such as the presence of atrial septal aneurysm, comorbidities, and stroke risk profiles.

Further research is warranted to address the observed heterogeneity and elucidate the long-term outcomes of PFO closure, particularly concerning atrial fibrillation management and its potential implications for recurrent stroke risk. Additionally, advancements in device technology and procedural techniques may help mitigate adverse events, enhancing the overall safety profile of PFO closure. Multiple meta-analyses, including those by Garg et al. and Ntaios et al. [19,22], have emphasized the importance of extended follow-up and patient-level analyses to enhance the understanding of risks and benefits across diverse patient populations. This work builds upon our previously published systematic review entitled "Comparison of Patent Foramen Ovale Closure vs Medical Therapy for the Prevention of Recurrent Cryptogenic Stroke" [23], which comprehensively identified and evaluated RCTs comparing PFO closure with medical therapy in patients with cryptogenic stroke. For the present update, we extended the literature search and re-analyzed the cumulative evidence.

Limitations

This meta-analysis has several limitations that must be considered when interpreting its findings. The primary limitation is the lack of individual-level data, which restricts the ability to perform nuanced analyses and identify patient-specific factors influencing treatment outcomes. Such data would enable a deeper exploration of how anatomical, clinical, and procedural variables interact to affect the efficacy and safety of PFO closure, providing stronger guidance for personalized treatment strategies.

Although most analyses demonstrated minimal heterogeneity and no evidence of publication bias, moderate heterogeneity in outcomes such as recurrent stroke prevention and atrial fibrillation incidence likely reflects variability in study populations, closure devices, and procedural techniques, underscoring the need for standardized protocols and larger, more diverse study cohorts to improve the reliability and relevance of future research. Previous meta-analyses, including those by Garg et al. and Ahmad et al. [19,20], have similarly emphasized the importance of extended follow-up and robust patient-level data to better understand risks and benefits across diverse populations.

The relatively small number of included studies (n = 7) further limits the generalizability of findings, despite subgroup analyses offering insights into high-risk populations like patients with atrial septal aneurysm or large shunt size. Future research should rigorously evaluate additional variables such as neuroimaging markers (e.g., diffusion-weighted MRI findings) or anatomical features like a Chiari network or giant Eustachian valve to refine patient selection for transcatheter PFO closure.

While this meta-analysis suggests that PFO closure is superior to medical therapy in preventing recurrent strokes among patients with cryptogenic stroke, this advantage does not extend to those with TIAs as qualifying events, highlighting the importance of comprehensive clinical assessments in guiding therapeutic decisions.

Finally, the lack of long-term follow-up data in several studies limits the understanding of the durability of benefits and risks associated with PFO closure, emphasizing the need for extended follow-up periods to determine whether the increased risk of atrial fibrillation translates into a higher long-term stroke risk or other adverse outcomes. These limitations collectively underscore the importance of further research incorporating individual-level data and exploring additional anatomical and clinical factors to refine treatment strategies and confirm these findings.

## Conclusions

Our meta-analysis revealed that while patients with PFO who underwent PFO closure experienced a lower incidence of cryptogenic stroke compared to those on medical therapy, they exhibited a higher prevalence of atrial fibrillation. These findings underscore the importance of weighing the benefits of reduced stroke risk against the increased likelihood of atrial fibrillation when considering PFO closure as a treatment option. Further research is necessary to investigate the significance of post-closure atrial fibrillation as well as the role of anticoagulation in selected patients with stroke and PFO.

## References

[1] Yaghi S, Bernstein RA, Passman R, Okin PM, Furie KL (2017). Cryptogenic stroke: research and practice. Circ Res.

[2] Sacco RL, Ellenberg JH, Mohr JP, Tatemichi TK, Hier DB, Price TR, Wolf PA (1989). Infarcts of undetermined cause: the NINCDS Stroke Data Bank. Ann Neurol.

[3] Meissner I, Whisnant JP, Khandheria BK (1999). Prevalence of potential risk factors for stroke assessed by transesophageal echocardiography and carotid ultrasonography: the SPARC study. Stroke Prevention: Assessment of Risk in a Community. Mayo Clin Proc.

[4] Meier B, Lock JE (2003). Contemporary management of patent foramen ovale. Circulation.

[5] Cheng T, Gonzalez JB, Testai FD (2021). Advances and ongoing controversies in PFO closure and cryptogenic stroke. Handb Clin Neurol.

[6] Handke M, Harloff A, Olschewski M, Hetzel A, Geibel A (2007). Patent foramen ovale and cryptogenic stroke in older patients. N Engl J Med.

[7] Overell JR, Bone I, Lees KR (2000). Interatrial septal abnormalities and stroke: a meta-analysis of case-control studies. Neurology.

[8] Bridges ND, Hellenbrand W, Latson L, Filiano J, Newburger JW, Lock JE (1992). Transcatheter closure of patent foramen ovale after presumed paradoxical embolism. Circulation.

[9] Li J, Liu J, Liu M, Zhang S, Hao Z, Zhang J, Zhang C (2015). Closure versus medical therapy for preventing recurrent stroke in patients with patent foramen ovale and a history of cryptogenic stroke or transient ischemic attack. Cochrane Database Syst Rev.

[10] Sitwala P, Khalid MF, Khattak F (2019). Percutaneous closure of patent foramen ovale in patients with cryptogenic stroke - an updated comprehensive meta-analysis. Cardiovasc Revasc Med.

[11] Liu Y, Wu Y, Xiong L (2021). Surgical vs. drug therapy in patients with patent foramen ovale and cryptogenic stroke. Herz.

[12] Sterne JA, Sutton AJ, Ioannidis JP (2011). Recommendations for examining and interpreting funnel plot asymmetry in meta-analyses of randomised controlled trials. BMJ.

[13] Mas JL, Derumeaux G, Guillon B (2017). Patent foramen ovale closure or anticoagulation vs. antiplatelets after stroke. N Engl J Med.

[14] Furlan AJ, Reisman M, Massaro J (2012). Closure or medical therapy for cryptogenic stroke with patent foramen ovale. N Engl J Med.

[15] Lee PH, Song JK, Kim JS (2018). Cryptogenic stroke and high-risk patent foramen ovale: the DEFENSE-PFO trial. J Am Coll Cardiol.

[16] Meier B, Kalesan B, Mattle HP (2013). Percutaneous closure of patent foramen ovale in cryptogenic embolism. N Engl J Med.

[17] Søndergaard L, Kasner SE, Rhodes JF (2017). Patent foramen ovale closure or antiplatelet therapy for cryptogenic stroke. N Engl J Med.

[18] Saver JL, Carroll JD, Thaler DE, Smalling RW, MacDonald LA, Marks DS, Tirschwell DL (2017). Long-term outcomes of patent foramen ovale closure or medical therapy after stroke. N Engl J Med.

[19] Garg A, Thawabi M, Rout A, Sossou C, Cohen M, Kostis JB (2019). Recurrent stroke reduction with patent foramen ovale closure versus medical therapy based on patent foramen ovale characteristics: a meta-analysis of randomized controlled trials. Cardiology.

[20] Ahmad Y, Howard JP, Arnold A (2018). Patent foramen ovale closure vs. medical therapy for cryptogenic stroke: a meta-analysis of randomized controlled trials. Eur Heart J.

[21] Pan X, Xu L, Zhou C, Zhang Z, Sun H (2021). Meta-analysis of patent foramen ovale closure versus medical therapy for prevention of recurrent ischemic neurological events: impact of medication type. Medicine (Baltimore).

[22] Ntaios G, Papavasileiou V, Sagris D, Makaritsis K, Vemmos K, Steiner T, Michel P (2018). Closure of patent foramen ovale versus medical therapy in patients with cryptogenic stroke or transient ischemic attack: updated systematic review and meta-analysis. Stroke.

[23] Hamodat O, Almuzainy S, Yahya R, Koniali S (2025). Comparison of patent foramen ovale closure vs medical therapy for the prevention of recurrent cryptogenic stroke: a systematic review. J Saudi Heart Assoc.

